# Energy-Reduced Arrhythmia Termination Using Global Photostimulation in Optogenetic Murine Hearts

**DOI:** 10.3389/fphys.2018.01651

**Published:** 2018-11-27

**Authors:** Raúl A. Quiñonez Uribe, Stefan Luther, Laura Diaz-Maue, Claudia Richter

**Affiliations:** ^1^RG Biomedical Physics, Max Planck Institute for Dynamics and Self-Organization, Göttingen, Germany; ^2^Institute for Nonlinear Dynamics, Georg-August University, Göttingen, Germany; ^3^Department of Pharmacology and Toxicology, University Medical Center, Göttingen, Germany; ^4^German Center for Cardiovascular Research (DZHK e.V.), Partner Site Göttingen, Göttingen, Germany; ^5^Department of Cardiology and Pneumology, University Medical Center, Göttingen, Germany

**Keywords:** optogenetics, energy-reduced defibrillation, cardiac arrhythmia, channelrhodopsin-2, photostimulation, global illumination

## Abstract

Complex spatiotemporal non-linearity as observed during cardiac arrhythmia strongly correlates with vortex-like excitation wavelengths and tissue characteristics. Therefore, the control of arrhythmic patterns requires fundamental understanding of dependencies between onset and perpetuation of arrhythmia and substrate instabilities. Available treatments, such as drug application or high-energy electrical shocks, are discussed for potential side effects resulting in prognosis worsening due to the lack of specificity and spatiotemporal precision. In contrast, cardiac optogenetics relies on light sensitive ion channels stimulated to trigger excitation of cardiomyocytes solely making use of the inner cell mechanisms. This enables low-energy, non-damaging optical control of cardiac excitation with high resolution. Recently, the capability of optogenetic cardioversion was shown in Channelrhodopsin-2 (ChR2) transgenic mice. But these studies used mainly structured and local illumination for cardiac stimulation. In addition, since optogenetic and electrical stimulus work on different principles to control the electrical activity of cardiac tissue, a better understanding of the phenomena behind optogenetic cardioversion is still needed. The present study aims to investigate global illumination with regard to parameter characterization and its potential for cardioversion. Our results show that by tuning the light intensity without exceeding 1.10 mW mm-2, a single pulse in the range of 10–1,000 ms is sufficient to reliably reset the heart into sinus rhythm. The combination of our panoramic low-intensity photostimulation with optical mapping techniques visualized wave collision resulting in annihilation as well as propagation perturbations as mechanisms leading to optogenetic cardioversion, which seem to base on other processes than electrical defibrillation. This study contributes to the understanding of the roles played by epicardial illumination, pulse duration and light intensity in optogenetic cardioversion, which are the main variables influencing cardiac optogenetic control, highlighting the advantages and insights of global stimulation. Therefore, the presented results can be modules in the design of novel illumination technologies with specific energy requirements on the way toward tissue-protective defibrillation techniques.

## 1. Introduction

Spatiotemporal dynamics in biological systems, particularly the control of complex excitation patterns, are a fundamental nonlinear problem with extensive potential in medical engineering and therapeutic application. Due to the intrinsic complexity of cardiac tissue, it is challenging to understand in detail the underlying biophysical mechanisms of arrhythmia. The normal sinus rhythm of the heart is triggered by regular, quasi-planar waves of electric depolarization. Spatiotemporally chaotic activation patterns have been identified, and are shown to be responsible for arrhythmic, life-threatening regimes (Davidenko et al., 1992; Luther et al., 2011; Christoph et al., 2018). The inferences of patterns in electrocardiograms (ECG), which showed up as irregular, sometimes aperiodic structures, gave the impulse to think of arrhythmia and especially of ventricular fibrillation as an uncontrolled, shivering activation of heart muscle. Thereby the underlying patterns are results of multiple erratic excitation waves changing in direction and shape. The complexity of wave patterns, leading to spatiotemporal chaotic regimes, is a consequence of the non-linearity. The dynamical processes are characterized by the annihilation of interacting waves, a mechanism also found in other physical systems (Panfilov and Holden, 1990; Jalifé et al., 1998). State-of-the-art therapies include high energy electrical shocks applied either external or internal to defibrillate the heart. These, for the patient mostly painful, shocks terminate the chaotic spreading activity almost certainly, but are suspected to worsen the existing tissue conditions mostly due to their potential electroporating effect on cardiomyocytes. Hence, they also serve as trigger for new arrhythmia with increasing probability over time. Much work has been devoted to the search for improved therapies (see e.g., Zipes and Jalife, 2009). Methods such as Anti-Tachycardia Pacing (ATP), already used in implantable devices, involve very small electrical currents delivered by a single electrode. Provided that the pacing frequency is high enough, ATP can terminate arrhythmia with a fairly high success rate (Wathen et al., 2004). Regardless, even with a high success rate the case of failure can never be disesteemed. Especially stationery vortex-activities are difficult to terminate with only one pacing electrode, which is not close enough to the pinning region. So it is not astonishing that several research groups are investigating advanced implementations of ATP compared to the traditional applied defibrillation shocks (Efimov et al., 2000; Exner, 2005). In addition to the ATP, the Low-Energy Anti-Fibrillation Pacing (LEAP) method was announced. It consists in pacing the tissue with an externally applied electric field. *In vitro* and *in vivo* experiments have provided ample evidence that LEAP significantly reduces the energy necessary to terminate atrial and ventricular fibrillation (Fenton et al., 2009; Luther et al., 2011) by using repeated stimulation with fields of lower amplitude. One crucial feature of LEAP is that it is based on multiple virtual electrodes induced by intrinsic obstacles. Referring to former *in vitro* and *in vivo* experiments (Exner, 2005), especially defibrillation approaches implementing multiple pacing sites have significant influence on arrhythmia specific excitation patterns resulting in rapid synchronization. Anyhow, in order to stimulate at multiple pacing sites either multiple implanted electrodes or specific electrical fields are necessary, which raises obvious translational hurdles. Also, all these valuable methods are still based on electrical shock application, which in turn can never be fully acquitted of potential worsening side effects. Consequentially, the evaluation of new cardiac treatments with side effect diminishing properties but fairly high success rates has to be brought into focus. At this point patterned light control of optogenetically modified cardiac tissue gives the opportunity to specifically stimulate well-defined tissue regions without critical Faraday reactions. Optogenetic photostimulation uses light of specific wavelengths to activate light-sensitive ion channels, which works without former electrically induced membrane potential changes (Bruegmann et al., 2011; Deisseroth, 2011). Recently, optogenetic cardioversion methods applying localized photostimulation were shown to be feasible (Zaglia et al., 2015; Bruegmann et al., 2016; Crocini et al., 2016; Nyns et al., 2016; Richter et al., 2016). Although much effort was put into the characterization of locally applied light intensity and energy (Bruegmann et al., 2010; Zaglia et al., 2015; Diaz-Maue et al., 2018) the underlying dependencies of light intensity, pulse duration and successful cardioversion remains somehow elusive.

However, recent studies showed that inducing multi-centered excitation within the arrhythmic tissue leads to a better control of spatiotemporal wave patterns, typical for fibrillation (Pumir et al., 2007; Luther et al., 2011; Janardhan et al., 2012). Having this in mind, successful global photostimulation would represent the maximum number of available pacing sites. In comparison with the conventional high-energetic electrotherapy, global photostimulation could overcome adverse side-effects like electroporation or unwanted co-stimulation of sensible neurons responsible for pain sensation during defibrillation. Indeed there still remain some questions to be solved before global illumination or multi-site photostimulation could count for reliable defibrillation. With regard to potential clinical translation especially the dependencies between the minimal required light intensity and pulse duration as well as the applied over-all energy are important for the design and optimization of implanteable light-emitting devices. Furthermore, the investigation of global photodefibrilation and the underlying spatiotemporal mechanisms could help to deepen our understanding of the mode of action of conventional electrotherapy.

In the present study, we determine the threshold value of the applied global photostimulation as a function of the intensity and pulse duration, and we compare our experimental results with other photostimulation data. Another main question is whether it is possible to terminate arrhythmia like patterns by light-induced excitation.

## 2. Materials and methods

All experiments were done in accordance with the current version of the German animal welfare law and were reported to our animal welfare representatives; the application for approval of animal experiments has been approved by the responsible animal welfare authority (Lower Saxony State Office for Consumer protection and Food Safety). Humane welfare-oriented procedures were carried out in accordance with the Guide for the Care and Use of Laboratory Animals and done after recommendations of the Federation of Laboratory Animal Science Associations (FELASA).

### 2.1. Langendorff perfusion

The presented experiments are based on retrograde Langendorff perfusion using a constitutive transgenic mouse model, α-MHC-ChR2, which restricts expression of channelrhodopsin-2 (ChR2) to cardiac tissue. The ChR2 expression was proven by biomolecular protocols. The perfusion protocol includes two different tyrode solutions, either for arrhythmia induction or maintenance. The maintenance tyrode composition was described elsewhere (Richter et al., 2016). Briefly, 130mM NaCl, 4 mM KCl, 1 mM MgCl 2, 24 mM NaHCO 3, 1.8 mM CaCl 2, 1.2 mM KH 2PO 4, 5.6 mM glucose, 1 % albumin/BSA were aerated with carbogen (95% oxygen and 5% CO 2).

#### 2.1.1. Arrhythmia induction

In order to induce sustained arrhythmia we lowered the concentration of KCl to 2 mM, so that the arrhythmia induction tyrode contains 130 mM NaCl, 2 mM KCl, 1 mM MgCl 2, 24 mM NaHCO 3, 1.8 mM CaCl 2, 1.2 mM KH 2PO 4, 5.6 mM glucose, 1 % albumin/BSA were aerated with carbogen (95 % oxygen and 5 % CO 2). Because of a reduction in transmural dispersion of repolarization the so induced hypokalemia enhances arrhythmia induction (Killeen et al., 2007). In addition, 100 μM Pinacidil, which is a KATPchannel activator, was applied to shorten the action potential duration (Wilde, 1994; Glukhov et al., 2010). The combination of both factors has been successfully applied to induce long sustained ventricular arrhythmia in murine Langendorff-perfused hearts (Bruegmann et al., 2016). Sustained arrhythmia was induced by applying 30 electrical pulses with a needle electrode in the range of 30–50 Hz. To diminish motion artifacts in optical records, the contraction uncoupling reagent Blebbistatin (*c* = 5 μM, Thermo Fisher Scientific) was administrated. Potientiometric staining was achieved by the red-shifted dye Di-4-ANBDQPQ (*c* = 50 μM, Thermo Fisher Scientific) via bolus injection. All perfusion experiments were done at 37 °C.

#### 2.1.2. Optical mapping

**Figure 1:**
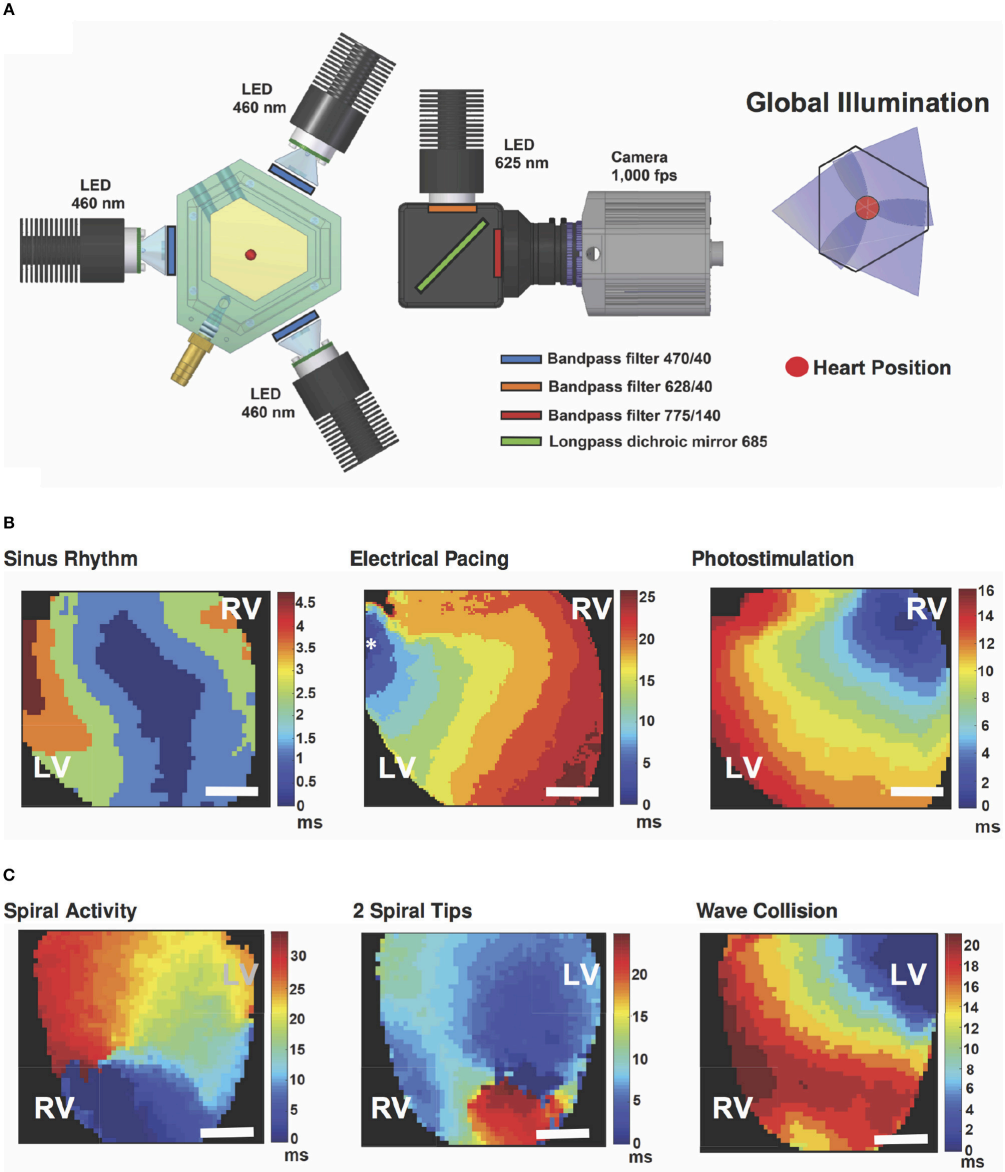
Global illumination and optical mapping setup. **(A)** The heart is perfused in a bath surrounded by three blue-LED for panoramic photostimulation. Light of a 625 nm LED is reflected via a dichroic mirror onto the heart surface. Emission was recorded through a 775±70 nm bandpass filter and an EMCCD camera at 1 kHz. Global illumination is reached via overlapping light cylinders. **(B)** Activation maps in posterior view of the heart of spontaneous sinus rhythm and stimulation examples electrically (electrode position is marked with an asterisk) as well as by global illumination. **(C)** Exemplary activation maps in anterior view of the heart of induced cardiac arrhythmia and collision of waves leading to annihilation of arrhythmic pattern. Color-bars indicate activation time in ms and the scale-bars mark 2 mm. A movie of the wave collision is available as (Supplementary Material Movie 1). LV - Left Ventricle, RV - Right Ventricle.

A longpass 685 nm dichroic mirror (FF685-Di02-25x36, Semrock) was integrated to reflect the excitation light from a 625 nm mounted LED (M625L3, Thorlabs) after a bandpass filter (FF02-628/40-25, Semrock) onto the heart. The emission light was collected with a 775 ± 70 nm bandpass filter (FF01-775/140-25, Semrock) before reaching the camera (see Figure 1). Epicardial signal recording of the anterior wall was done with an electron multiplying charged coupled device (EMCCD, Cascade 128+, Photometrics) camera with a spatial resolution of 64 × 64 pixels (133 μm per pixel) at 1 kHz. Camera control was achieved using custom-made recording software and the electrical heart activity was recorded using a monophasic action potential (MAP) electrode (BIOPAC Systems, Inc.).

### 2.2. Optogenetic illumination strategies

We employed local and global illumination to stimulate the heart, whereby only global illumination was used for cardioversion. Local illumination was achieved by positioning the tip of an optical fiber of ⌀ = 400 μm in contact with the left ventricle. On the other hand, in order to achieve a consistent optogenetic stimulation of the whole heart surface and therewith global illumination, the hearts were vertically arranged surrounded by three blue-light emitting diodes (blue-LED, Thorlabs) with their wavelengths centered at 460 nm and limited by a 470 ± 20 nm bandpass filter (ET470/40x, Chroma) (see Figure 1). Synchronous millisecond control of LED at different intensities was conducted via a function generator (Arbitrary Function Generator A2230, Agilent Instruments). Intensity measurements were done using the PM100D optical power meter and the S120VC photodiode power sensor (Thorlabs). Since the experimental setup consists of three blue-LED spaced at 120°, the intensity was measured directly facing each LED from the heart position and the calculated mean was considered the overall light intensity during global illumination.

To minimize effects of potential edema as well as metabolic changes during repeated arrhythmia periods on the defibrillation success rate, we limited the experimental time to 2 h and a maximum number of defibrillation attempts of 50.

### 2.3. Data analysis

The obtained fluorescent images were analyzed and processed using MatLab (MathWork, Inc.). Briefly, spatial and temporal smoothing filter were applied after pixelwise normalization. Overlapping of the blue light with the emission signal of the dye was removed by subtracting the average difference in intensities with and without blue light from each pixel over time (see Figure S1). The estimation of the total surface area of both ventricles and atria was achieved by reconstructing three-dimensional heart shapes from photographs using a shape from contour approach as previously described (Christoph et al., 2017). In total three hearts were used to calculate an average epicardiac surface area of 274 mm2. Statistical analysis was done by Student's *t*-test (one-tailored and unequal variance) comparing each increasing step with the following value. Throughout the text results are indicated with ± standard deviation, unless otherwise noted.

Concerning local and global pacing experiments a train of 20 pulses was applied, whereby only the last 10 pulses were considered for the analysis. The minimum pulse lengths needed to reach 1:1 capture in all the tested hearts for different intensities were investigated. Each light intensity was tested in combination with maximal four different pulse duration values.

To determine the success rate of arrhythmia termination, we induced multiple arrhythmia in each heart and attempted termination 10 times with each light intensity-duration combination. To consider an arrhythmia as sustained we waited for 5 s after induction before attempting termination, which consisted of illuminating the whole heart by simultaneously turning on the three LED for the duration and intensity tested. If the arrhythmia stopped within maximum 1 s after the conclusion of stimulation, it was considered a successful attempt. In case of failed termination, we applied backup defibrillation, which consisted on increasing the intensity and duration of the pulses. To support the defibrillation procedure the hearts were perfused with maintenance tyrode (as described in section 2.1 and 2.1.1). The parameter combination with the highest termination rate and the lowest pulse duration values were determined as the most efficient ones. They also served as a decision-critical point for parameter change.

## 3. Results

### 3.1. Global vs. local optogenetic pacing

To characterize successful cardiac photostimulation, we tested the necessary pacing parameters to achieve 1:1 capture with respect to different pulse durations and light intensities. In order to avoid potential photochemical reactions and other side-effects stimulation light was turned off after every pacing.

**Figure 2:**
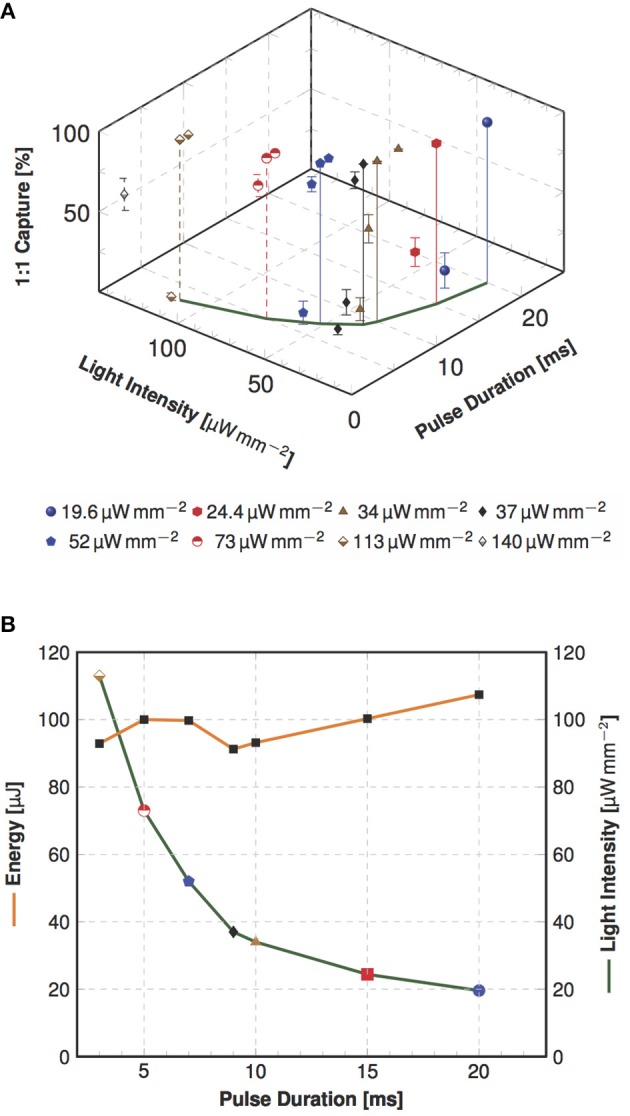
Dependency of pulse duration and light intensity as well as pulse energy in global optogenetic pacing. **(A)** The three-dimensional plot illustrates the averaged success rate ± SEM of 1:1 capture in dependency of investigated combinations of light intensity and pulse duration (as indicated in Table 1). In this view the variation of measured capture rates is clearly visible, but still approving the dependency of successful 1:1 capture of light intensity and pulse duration. In the x-y section are the most efficient combinations marked, which is visualized in a plain view in the next panel. **(B)** Shown are the most efficient combinations of required light intensity and energy for different pulse duration values (*N* = 6). The analysis shows a nearly constant energy, but an increase in intensity when the pulse duration is shortened.

First, global pacing was applied. Table 1 gives an overview of the step-wise measured intensities in relation to pulse duration and success rate. Our results prove that a shorter pulse duration correlates with higher intensities needed to gain 1:1 capture. The shortest pulse duration to successfully pace was *t*_*gpacing*_ = 3 ms applying an intensity of *I*_*gpacing*_ = 113 μW mm-2 (*N* = 6). For pacing with 20 ms pulses successful pacing required an intensity of *I*_*gpacing*_ = 19.6 μW mm-2. Thus, constituting the lowest intensity reported to pace an optogenetic murine heart by photostimulation so far. Pacing the heart with *I*_*gpacing*_ = 140 μW mm-2 was also tested for *t*_*gpacing*_ = 2 ms, but could only be considered successful in one heart. Figure 2 shows the intensities and energies necessary to pace the heart with 1:1 capture at different pulse lengths using global stimulation. The graph shows that the energy necessary to pace at different intensity and duration combinations remained constant at an average of *E*_*gpacing*_ = 98 ± 5 μJ.

**Table 1:** Measured pulse duration and light intensity combinations.

		**Light intensity [**μ**W mm**-2**]**							

		19.6	24.4	34	37	52	73	113	140
**Pulse duration [ms]**	20	100 ± 0							
	15	23 ± 11	100 ± 0						
	12.5		40 ± 8.9	100 ± 0					
	10			100 ± 0					
	9			61 ± 8.8	100 ± 0				
	8			14 ± 7.1	93 ± 5.2	100 ± 0			
	7				20 ± 7.9	100 ± 0			
	6				6.5 ± 3.6	90 ± 4.7	100 ± 0		
	5					13 ± 7.2	100 ± 0		
	4						86 ± 6.9	100 ± 0	
	3							100 ± 0	
	2							5.2 ± 2.3	57 ± 10

*Inscribed is the averaged success rate [%] ± SEM of 1:1 capture during global illumination. The visible trend within the success rate regarding the dependency of light intensity and pulse duration is also mirrored in a constant energy level. Highlighted values indicate the most efficient pulse duration and light intensity combinations, which are drawn in Figure 2B together with the corresponding energy values. The shown values are based on 23 measurements from N = 6 hearts*.

For comparison, we also conducted local photostimulation. Considering an increase in intensity required to pace the heart when stimulating smaller areas, we measured the intensity necessary to achieve 1:1 capture using an optical fiber of *A*_*fiber*_ = 0.126 mm2 with a *t*_*lpacing*_ of 3 ms, 7 ms, 9 ms and 15 ms. Compared to global pacing, all the intensity values required to obtain 1:1 capture were increased by minimum one order of magnitude with a maximum of *I*_*lpacing*_ = 1.77 mW mm-2.

In the course of these experiments, it could be observed that the average energy delivered to the epicardium is constant for both global and local illumination. In spite of the clear increase in intensity, it is in average 30-fold lower when pacing locally than globally, at *E*_*lpacing*_ = 2.8 ± 0.6 μJ and *E*_*gpacing*_ = 98 ± 5 μJ, respectively.

### 3.2. Global optogenetic cardioversion

**Figure 3:**
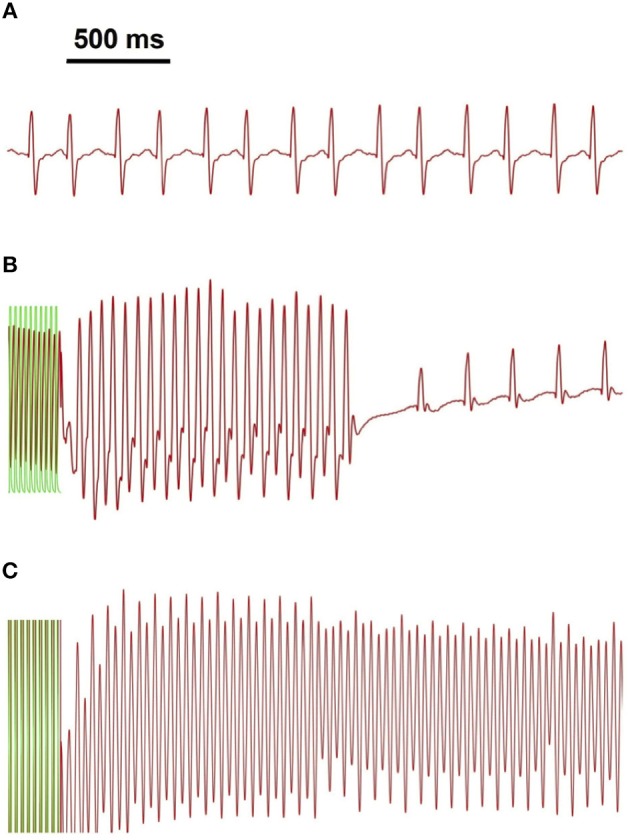
Arrhythmia classification on the basis of electrical signal analysis. Shown are exemplary traces of MAP measurements (in red) for **(A)** sinus rhythm, **(B)** non-sustained and **(C)** sustained arrhythmia. Green overlay indicates part of the electrical pacing used to induce arrhythmia.

In order to find the optimal parameters for optical cardioversion, we induced cardiac tachyarrhythmia applying Pinacidil and rapid electrical pacing. Therewith 75 % of all arrhythmia lasted more than *t*_*arr*_ = 5 s (59 out of 79 induced arrhythmia). We considered the *t*_*arr*_ = 5 s duration as threshold for classification as sustained, since about 90 % lasted for *t*_*arr*_ ≥ 10 s. Non-sustained arrhythmia shorter than the threshold lasted on average *t*_*arr*_ = 1.5 ± 1.0 s (Figure 3). The average arrhythmia frequency was *f*_*arr*_ = 24 ± 5 Hz, and one third of all induced arrhythmia lasted *t*_*arr*_ ≥ 60 s. Optical Mapping data showed multiple vortex-like wave dynamics, hence proving the successful induction of ventricular arrhythmia (Figure 1C).

#### 3.2.1. Optical parameter characterization

**Figure 4:**
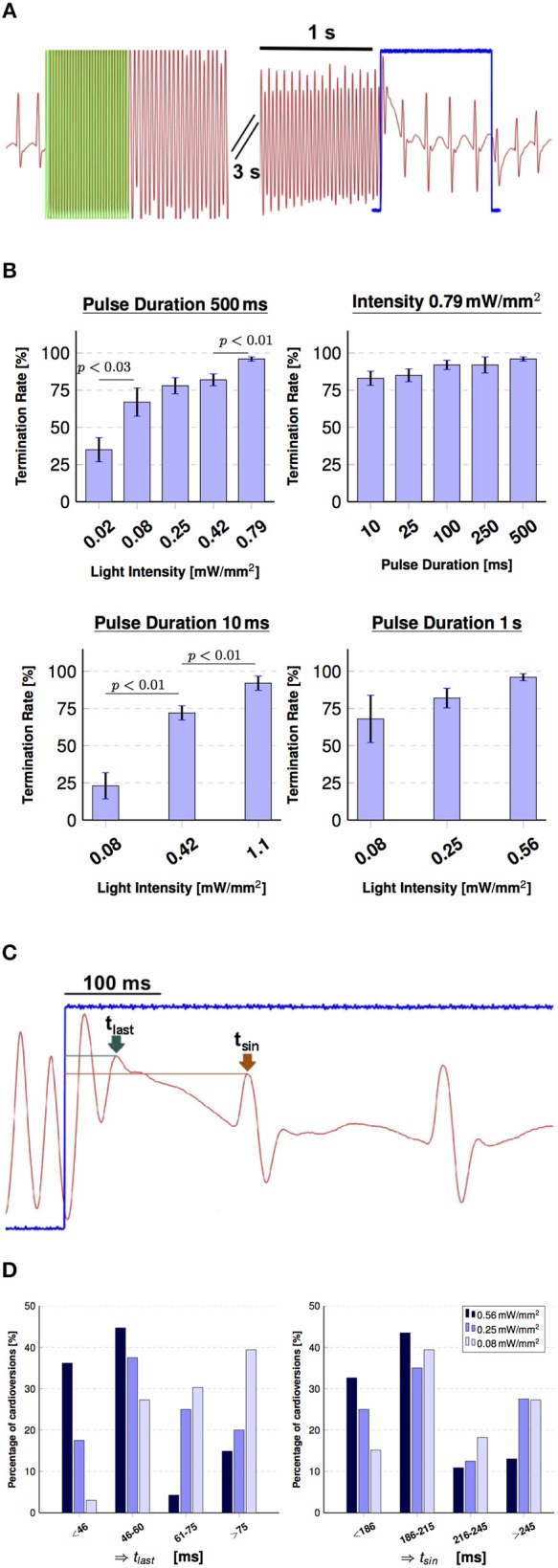
Global illumination for cardioversion. **(A)** Shows the pseudoECG signal during arrhythmia evocation via electrical pacing and termination using global illumination (green overlay indicates electrical pacing at 50 Hz, 30 pulse). The arrhythmic conditions were terminated with a 1 s stimulation pulse (indicated in blue), during which the normal sinus rhythm already returned. **(B)** Summary of the influence of intensity and pulse duration on cardioversion attempts. Successful optogenetic defibrillation rates (percentage of successful attempts reported as mean ± SEM, *N* = 6). **(C)** Visualizes the effect of the termination pulse shown in **(A)** in more detail. Two time points were defined in order to characterize the moment of arrhythmia termination for the 1 s pulses: *t*_*last*_ (green arrow), which denotes the last peak of the arrhythmia and *t*_*sin*_ (brown arrow), which denotes the first peak of the sinus rhythm during optogenetic stimulation. **(D)** Charts indicating the effect of intensity on cardioversion times in percentage of arrhythmia terminated in those periods of *t*_*last*_ (left) and *t*_*sin*_ (right) for 0.56 mW mm-2 (*N* = 47), 0.25 mW mm-2 (*N* = 40), 0.08 mW mm-2 (*N* = 33).

Our measurements concerning global optogenetic photo-defibrillation were triggered by Bruegmann et al. (2016), where local illumination cardioversion was first achieved by using an intensity of *I*_*cv*(*Brueg*.)_ = 0.40 mW mm-2 comprising a surface area of *A*_*heart*(*Brueg*.)_ = 143 mm2 with a 1 s pulse. In order to match the postulated amount of energy delivered by Bruegmann et al. [*E*_*cv*(*Brueg*.)_ = 57.2 mJ] with our global illumination, which spans a larger epicardial surface (*A*_*heart*_ = 274 mm2), we modulated the pulse duration by keeping the intensity constant at *I*_*cv*_ = 0.42 mW mm-2. Figure 4 shows the main results of the photo-defibrillation attempts. One single long stimulation pulse led to a successful arrhythmia termination in 82 % of the experiments, which is comparable to the results obtained by Bruegmann et al.

Subsequently, we assessed the influence of light intensity by maintaining the pulse duration constant at *t*_*cv*_ = 500 ms. While all tested light intensities successfully terminated the arrhythmia, a decrease in light intensity correlated with a significant depression in cardioversion efficiency (Figure 4B). At the most efficient light intensity of *I*_*cv*_ = 0.79 mW mm-2, a success rate of 96±2 % [mean ± standard error of the mean (SEM)] was achieved. However, a decrease in intensity by 40-fold still managed to revert the heart rhythm in more than 30 % of the attempts for the hearts tested (*N* = 6).

Afterwards, to evaluate the effect of pulse duration we kept the light intensity constant at *I*_*cv*_ = 0.79 mW mm-2 and applied pulses shorter than *t*_*cv*_ = 500 ms. However, no significant change in termination rate was found for pulse durations of 500, 250, 100, and 10 ms. A shortening of pulse duration from 500 to 10 ms resulted in a decline of 13 % of arrhythmia terminated (Figure 4B). Supposing the parameter combination of *t*_*cv*_ = 10 ms and *I*_*cv*_ = 0.79 mW mm-2 is as efficient as *t*_*cv*_ = 500 ms at the same light intensity, in the following we tested a constant pulse duration of *t*_*cv*_ = 10 ms while step-wise increasing light intensity from *I*_*cv*_ = 0.08 to 1.1 mWmm^−2^. Thereby we obtained successful termination in 92 ± 4 % of the experiments. Pursuing a wider comprehension of the effects of intensity and duration on global light-induced arrhythmia termination, we then experimented with 1 s long pulses, where an efficiency of 96 ± 2 % was achieved with *I*_*cv*_ = 0.56 mW mm-2.

### 3.3. Effects of global illumination on arrhythmia patterns

Since the 1 s stimuli lasts multiple tachycardia and sinus rhythm cycles, we identified that the transition from an arrhythmic state to the natural sinus frequency took place at different time points from the onset of stimulation. The electrical signal shown in Figures 4A,C shows a clear sinus activity even before the photostimulation has ended. To estimate the time between the beginning of stimulation and the moment of optical cardioversion, we defined two characteristic points in time of the MAP signal (Figure 4C). First we define *t*_*last*_ as the moment when the last peak of the arrhythmia occurred, and second *t*_*sin*_ as the time point when the sinus rhythm signal first appeared. These two values were calculated for all the successful arrhythmia terminations of different intensities for the 1 s pulses. Figure 4D illustrates the percentages of *t*_*last*_ and *t*_*sin*_ for four time lapses. it can be observed that *t*_*last*_ occurred at ≤ 60 ms of stimulus for 81 % of the cardioversions when triggered with *I*_*cv*_ = 0.56 mW mm-2. However, this seems to depend on the light intensity, since cardioversions triggered with *I*_*cv*_ = 0.25 mW mm-2 showed this phenomenon in 55 % of the cases and with an intensity of *I*_*cv*_ = 0.08 mW mm-2 in only 30 %. Furthermore, *t*_*sin*_ ≤ 215 ms occurred in 76, 60, and 54% of the respective tested intensities.

#### 3.3.1. Cardiac dynamics during global illumination

**Figure 5:**
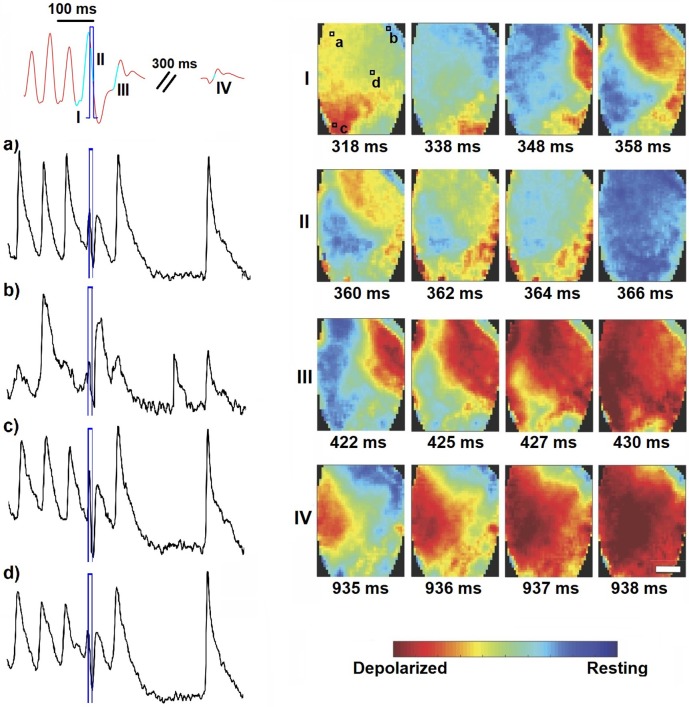
Wave dynamics during short global light pulse (10 ms, 0.25 mW mm-2, highlighted in blue). Shown are the MAP recording (in red) and optical traces of several local spots on the ventricles (in black, as indicated in panel I). Optical mapping analysis revealed vortex-like electrical activity on the heart surface (image sequence I), which is disturbed by global illumination (image sequence II) and thus provoking wave collision resulting in annihilation (image sequence III). Sinus activity follows after a peak corresponding to depolarization of the whole heart (image sequence IV). The corresponding movie is available as (Supplementary Material Movie 2).

**Figure 6:**
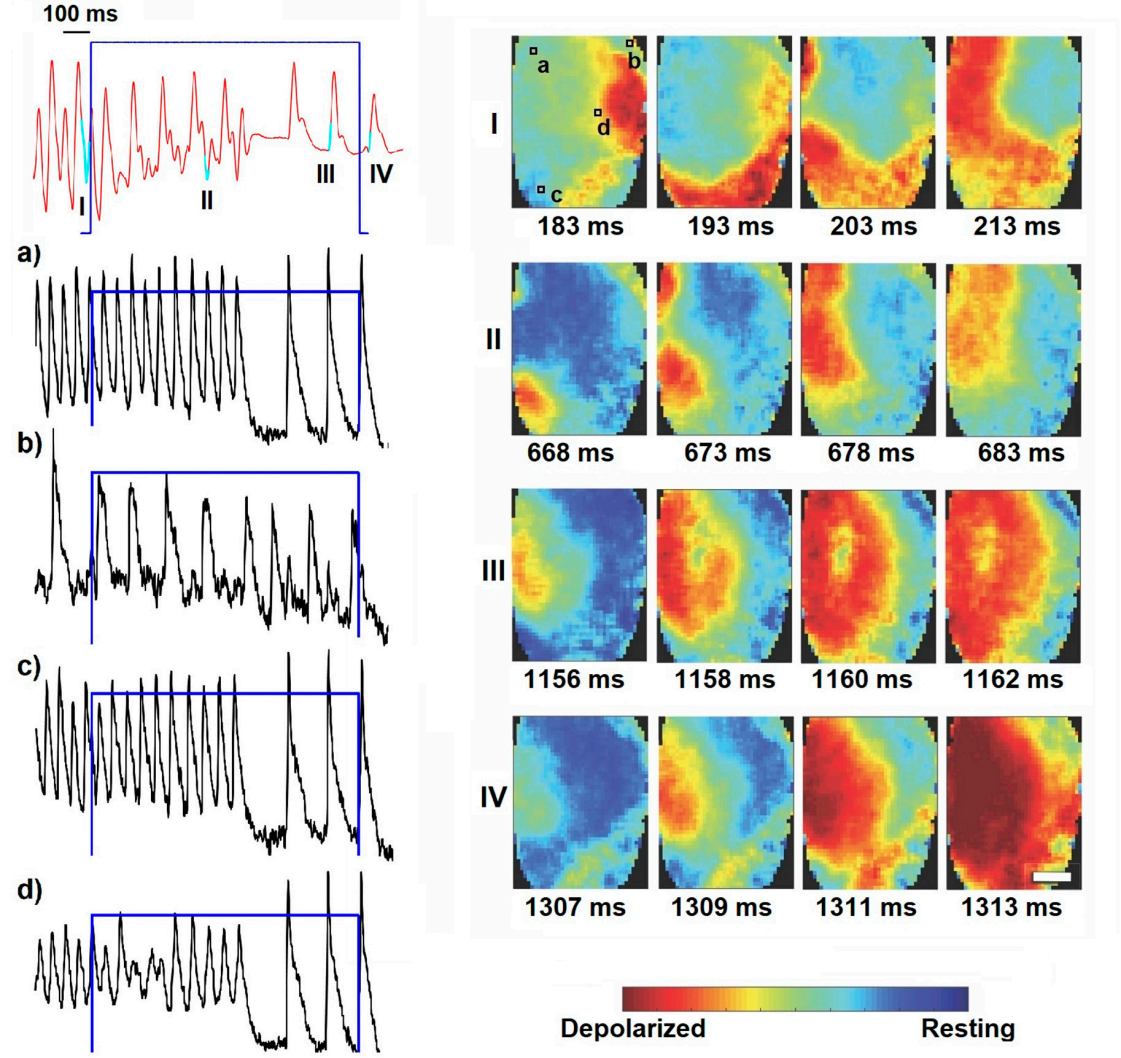
Optical determination of the second identified mechanism, which was only observed in 1 s pulses (0.25 mW mm-2, highlighted in blue). Here the locally stable spiral activity (image sequence I) is disrupted by the illumination resulting in extrusion of the arrhythmic wave pattern to the boundaries (image sequence II). Already during photostimulation the sinus rhythm resets (image sequence III) and continues after switching off (image sequence IV). The scale-bars indicate 2 mm and are equal for all images in the corresponding panel. Shown are the MAP recording (in red) and optical traces of several local spots on the ventricles (in black, as indicated in panel I). The corresponding movie is available as (Supplementary Material Movie 3).

The optical mapping analysis of the 10 ms as well as 1 s stimulation experiments combined with the analysis of *t*_*last*_ in 1 s pulse attempts led to identify two preferential mechanisms for global optical cardioversion. The first mechanism consists of a cardioversion that happens on the onset of illumination, mainly annihilating the spiral by the depolarization and following refractoriness of the cardiac tissue (Figure 5). While in the second mechanism, an unpinning and disturbance of the spiral can be observed (Figure 6), with the elimination of the spiral taking place at the mid and late stimulation period. Optical mapping data showing the two mechanisms could be found in the (Supplementary Material Movies 2, 3). As the dominant mechanism the annihilation was observed for both pulse duration 10 ms and 1 s, and took place in 90 % of all cardioversions using 1 s pulses (*N* = 120). This mechanism included a *t*_*last*_ = 58 ± 19 ms. In contrast, the second mechanism was only observed in the 1 s pulses with an averaged *t*_*last*_ = 296 ± 122 ms. Analyzing the MAP recordings led to a clear distinction between the two mechanisms, whereby the number of arrhythmic excitation peaks during *t*_*last*_ was ≤ 2 for 86 % of all the annihilation observations, while all the unpinning cases presented > 4 arrhythmia cycles during *t*_*last*_.

## 4. Discussion

The control of spatiotemporal cardiac regimes and the influence of the thereto applied electrical pulses have been investigated extensively in theory and experiment in the last decades (see e.g., Pumir and Krinsky, 1999; Takagi et al., 2004). In consequence, especially the diminishment of adverse side effects of high-energy electrical shocks, like e.g., electroporatic cell membrane disturbances, while keeping a very high success rate has been highly prioritized. In this manner, Janardhan et al. introduced a low-energy defibrillation approach to successfully terminate ventricular tachycardia (VT) applying multistage organized shocks (Janardhan et al., 2012). Thereby they eliminated the phase dependence of shock application, which is crucial for single shock therapy in VT. Furthermore, the possibility to control cardiac excitation dynamics by applying multi-centered new activation origins was proposed as one auspicious approach (Pumir et al., 2007; Luther et al., 2011). Luther et al. impressively showed that recruiting multiple pacing sites has remarkable success in counter-steering arrhythmic regimes, which in return makes tissue protective defibrillation feasible. Against this background, non-electrical approaches would benefit both the development of tissue protective defibrillation and the investigation of mechanistic associations without unpredictable worsening side effects. Cardiac optogenetics with its light initiated depolarization and accordingly hyperpolarization fulfills this position as a suitable tool to characterize mechanisms underlying multi-site pacing. Considering the complex non-linear dynamics of cardiac arrhythmia, photostimulation convinces with the highly controllable temporal as well as spatial resolution. Compared to conventional electrical approaches, the direct interaction of light with optogenetic cardiac tissue can trigger excitation in single cardiomyocytes or united cell structures without activating the surrounding tissue structures or inducing critical Faraday reactions (Ambrosi and Entcheva, 2014). Here, we presented a setup to investigate arrhythmia termination using global illumination. The feasibility of photon initiated cardioversion was shown recently by Bruegmann et al. (2016), Crocini et al. (2016), and Nyns et al. (2016). Concentrating mainly on low-numbered multi-site pacing Crocini et al. implemented a mechanistic approach using pulse series of an arrhythmia-specific light pattern that enabled cardioversion using a total amount of 1.8 mJ. In comparison the required light intensities up to 40 mW mm-2 exceeded the highest intensity implemented in our experiments by more than the 30-fold. Furthermore, Bruegmann et al. described an illumination protocol applying 1 % of the light intensity used by Crocini et al., but with a highly increased pulse duration of four pulses of 1 s with 1–5 s in-between accounting for a total amount of energy of 228.8 mJ. Having this in mind, in our experiments we concentrated on the characterization of the separate parameter combinations. In order to do so, the global stimulation of the whole cardiac tissue represents the maximum number of pacing sites available and thus it is comparable to conventional high-energy electrical defibrillation approaches. We showed the impact of illuminating the epicardial surface by pacing with the lowest intensities published so far. An interesting result is that the energy required to pace both globally and locally remained constant for each of the methods (98 ± 5 μJ and 2.8 ± 0.6 μJ, respectively) and increased with a growing stimulation area. Certainly, the excitation origin depends on different factors such as wavelength or light propagation characteristics. An interesting observation during global photostimulation is the correlation between increasing light intensity and rising efficiency of cardioversion. Lilienkamp et al. introduced *in silico* results similar to our experimental findings, which can give hints that the overall size of an excitable medium has a direct effect on the lifetime of chaotic spatiotemporal dynamics, like the ones seen during arrhythmia (Lilienkamp et al., 2017). One possible explanation could be the change in penetration depth when using higher light intensities for photostimulation, hence depolarizing a greater number of cells and creating a thicker reversible conduction pattern. This was also described by Watanabe et al. in experiments with ventricular slices, where they showed a temporal decrease on the effective size available for the arrhythmia to wander (Watanabe et al., 2017). In their work, Watanabe et al. proved that by increasing the transmurality of illumination, the chances of terminating an anatomical re-entry on the slices increased. This effect could be lead back to the indispensable fact of reaching the critical cell number for excitation evocation (Zipes et al., 1975). In contrast, a varied pulse duration at constant light intensity might prolong the depolarization of the stimulated layers of cardiac tissue without effectively changing the size of the stimulated cardiac tissue (Bruegmann et al., 2010).

Optogenetic intensity-dependent effects have been recorded at the level of single cells, quasi-two dimensional cell cultures and organs (Bruegmann et al., 2010; Nussinovitch et al., 2014; Burton et al., 2015; Nussinovitch and Gepstein, 2015; Zaglia et al., 2015). Accordingly, the effect of light intensity as described by *t*_*last*_ for the 1 s pulses could be explained by a theoretical combination of the amount of cells excited, the applied photocurrents and the conduction velocity of a generated excitation wave (Bruegmann et al., 2010; Burton et al., 2015; Zaglia et al., 2015). Moreover, the evidence showing that the majority of defibrillations took place during the onset of stimulation could be explained by the Channelrhodopsin-2 kinetics, since its photo-current reaches a peak during the first milliseconds of activation before dropping to a smaller current during continuous illumination (Nagel et al., 2003; Bruegmann et al., 2010). Yet, the difference on *t*_*sin*_ for each intensity is not likewise obvious, since it could be more related to the intrinsic sinus period of the heart than to the light induced effect on cardioversion. On the other hand, the effect of the pulse duration, even when the difference seems not to be significant might lead to diverse mechanisms of termination. Presumably, spiral disturbance is unlikely to happen during short pulses of 10 and 25 ms. Nevertheless, a longer pulse duration such as 500 ms or 1 s can still disrupt the tachycardia even after the onset of the stimulus, leading to a higher success rate. According to the biophysics of ChR2, these light-activated channelrhodopsins are un-selective for different cations like Ca^2+^ or Na^+^ (Schneider et al., 2015). Due to that fact, long photostimulation pulses probably lead to an accumulation of action potential relevant cations causing continuous depolarization to less negative membrane voltages. This assumption is supported by the fact that 10 % of cardioversions for 1 s stimulus occurred during mid-late stimulation.

However, the results presented here strengthen the application of cardiac optogenetics, although some underlying mechanisms still remain to be part of ongoing research. Especially the investigation of defibrillation protocols consisting of multistage anti-fibrillation pacing as well as multi-site pacing strategies have to be addressed, since such experiments would help to understand the dependencies of phase-specific pacing and success rate of arrhythmia termination. Therefore, all *ex vivo* experiments and the basic classification of photostimulation parameter function as a trigger for development, optimization and evaluation of a controllable as well as flexible light induced cardiac rhythm management. As a positive effect also the design of new non-electrical multi-site pacing methods with high potential of translation into clinically relevant approaches comes within reach.

### 4.1. Limitations

With the translation into *in vivo* conditions in mind, one has to mention that the *ex vivo* measurements lack the interference of blood specific absorption of ChR2 exciting wavelengths. Consequently, any effects during blue-light photostimulation *in vivo* would represent only a superficial excitation. Certainly, the cardiac excitation conduction is not restricted to epicardial phenomena but also connected to complex transmural wave propagation (Christoph et al., 2018). Therefore, near infrared modulated opsins would support the translation of *ex vivo* experimental results into clinically relevant *in vivo* attempts (Karathanos et al., 2016). Here we successfully achieved cardioversion using single pulses of divers pulse duration by tuning light intensities between 0.56 and 1.1 mWmm^−2^, delivering total energies 3–153.6 mJ to the epicardium. The importance of this low-intensity, pulse duration versatility relies on the fast spatiotemporal dynamics of arrhythmia, where the optimal duration of the stimulus could be arrhythmia-specific dependent. That would also result in a smaller amount of light being delivered to the cardiomyocytes located transmurally. The more precise estimation of excited cells within the ventricular wall and the characterization of light propagation through cardiac tissue are subject of current research projects. In the presented study we only examined murine hearts, for which reason the presented parameter findings can solely be considered as species specific. For larger species with much thicker ventricular walls or fatty epicardial regions the light intensity actually impacting light-sensitive cardiomyocytes will differ significantly. However, the gotten insights and general conclusions, like the fact that illuminating the whole epicardial surface will result in a decrease of minimum light intensity should be accountable also for larger species.

Furthermore, induced photochemical reactions during illumination are possible sources of direct cell response (Lubart et al., 2007). Lubart et al. showed that especially visible light (400 nm to 700 nm) could stimulate photobiomodulation and photosensitization in cardiomyocytes. These processes are inter alia initiated by generation of reactive oxygen species, which in return can lead to a change of the redox state of the cardiac cell and thus have indirect influence on the calcium induced calcium release (CICR) via redox sensitive L-type calcium channels. Although, compared to the energy used by Lubart et al. with 3.6 J cm-2 the total energy applied for global illumination in this study was 0.01–0.61 Jcm^−2^, which may have alleviated photochemical effects. Hence, the measurement and characterization of potential photobiomodulation with regard to repeated rapid photostimulation are considered for ongoing projects.

Experiments including optical mapping were done with Blebbistatin, which reduces motion artifacts due to its electromechanical uncoupling function. Since we used stimulation light of a blue wavelength (470 nm) it is important to mention, that the uncoupling effects of Blebbistatin could be reversed by blue light illumination (Fedorov et al., 2012). In our experiments we did not observe such light induced effects on the motion inhibition, which could be related to the fact that Fedorov et al. used much shorter wavelengths for illumination than we applied. This observation was also published by Swift et al. (2012). Nevertheless, it should be emphasized that Blebbistatin does possibly alter the metabolic state of ischemic cardiomyocytes (Swift et al., 2012).

## 5. Conclusion

According to recent cardiac optogenetic studies the usage of photodefibrillation seems to be feasible (Bruegmann et al., 2016, Crocini et al., 2016; Richter et al., 2016). Here we investigated for the first time the effects of global optogenetic epicardial illumination which benefits from the larger area covered to decrease the intensity required to stimulate the heart. In consequence, we managed to terminate arrhythmic excitation patterns using pulse lengths spanning three orders of magnitude, demonstrating an efficient and versatile low-intensity and low-energy method to investigate arrhythmia dynamics and manipulation. Besides, we observed two different mechanisms leading to optogenetic cardioversion, which exhibit not the same behavior than electrical cardioversion.

## Author contributions

RQ performed the experiments and analyzed the data. RQ and CR designed research, experiments and wrote the paper. SL and LD-M contributed to the discussion and edited the manuscript. All authors read and approved the manuscript.

### Conflict of interest statement

The authors declare that the research was conducted in the absence of any commercial or financial relationships that could be construed as a potential conflict of interest.
