# Supplementary material for: Energy-Reduced Arrhythmia Termination Using Global Photostimulation in Optogenetic Murine Hearts
Source: Front Physiol. 2018 Nov 27;9:1651. doi: 10.3389/fphys.2018.01651 (PMC6277892; doi:10.3389/fphys.2018.01651)
Supplement: Supplementary file 4 [file Data_Sheet_1.PDF]

# *Supplementary Material*: Energy-Reduced Arrhythmia Termination Using Global Photostimulation in Optogenetic Murine Hearts

## 1 SUPPLEMENTARY MOVIE CAPTIONS

**Movie 1.** Wave collision. Visualized is a collision of excitation waves leading to annihilation of the arrhythmic pattern.

**Movie 2.** Successfull light-induced cardioversion. The movie shows an arrhythmia termination mechanism, which was only observed in global illumination of 1 s pulse duration. The vortex-like excitation is disrupted by the photostimulation and pushed to the boundaries. The sinus rhythm resets already during photostimulation. Photostimulation is marked by a blue square in the lower right corner.

**Movie 3.** Arrhythmic excitation wave pattern and light-induced cardioversion. The movie visualizes a sustained ventricular arrhythmia, which was treated with global illumination of 10 ms pulse duration. Light-induced cardioversion sets in on the onset of illumination, causing wave collision and afterwards annihilation of arrhythmic waves. The normal sinus rhythm follows after whole heart depolarization. Global illumination is indicated by a blue square in the lower left corner.

## 2 SUPPLEMENTARY FIGURES

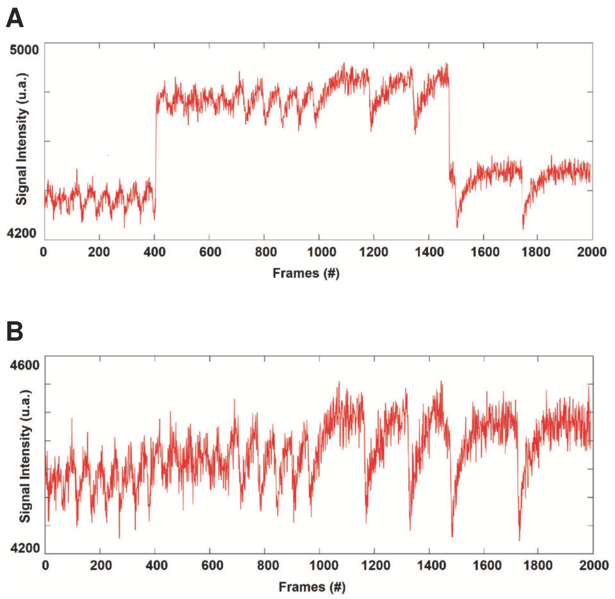


**Figure S1.** Crosstalk of stimulation light and fluorescence signal. The graphs show an exemplary time **(A)** before and **(B)** after removing the overlapping signals of stimulation light and fluorescence of the membrane voltage. Therefore, we substracted the average difference in intensities with and without stimulation light from each pixel.

## 3 SUPPLEMENTARY INFORMATION ON MOUSE MODEL

For this study we used a mouse model, which was generated by cooperation partners within the European research project EUTrigTreat (European Community's Seventh Framework Programme FP7/2007-2013 under grant agreement number HEALTH-F2-2009-241526.). While the new $\alpha$-MHC-ChR2 transgenic model will be reported in detail separately, in short these mice reproduce normally and without any apparent abnormalities as judged by echocardiography, electrocardiogram, and histology. Furthermore, the mouse hearts from 14-24 week old animals showed normal action potential spread similar to wild-type animals of the same age.
For confirmation of ChR2 expression we routinely perform polymerase chain reaction (PCR) of biopsy materials. The primer were purchased from Thermo Fisher Scientific and consist of the following sequences:

E091.4 5'-CTGCACTCTGTGGCCAGTAG-3'
9027-MHC 5'-TCCTGGTGGGAGAGCCATAG-3'
